# Natural Killer Cells from the Subcutaneous Adipose Tissue Underexpress the NKp30 and NKp44 in Obese Persons and Are Less Active against Major Histocompatibility Complex Class I Non-Expressing Neoplastic Cells

**DOI:** 10.3389/fimmu.2017.01486

**Published:** 2017-11-07

**Authors:** Alireza Shoae-Hassani, Maryam Behfar, Seyed Abdolreza Mortazavi-Tabatabaei, Jafar Ai, Rashin Mohseni, Amir Ali Hamidieh

**Affiliations:** ^1^Applied Cell Sciences and Tissue Engineering Department, School of Advanced Technologies in Medicine, Tehran University of Medical Sciences, Tehran, Iran; ^2^Proteome Research Center, Shahid Beheshti University of Medical Sciences, Tehran, Iran; ^3^Department of Pediatric Stem Cell Transplantation, Children’s Medical Center, Tehran University of Medical Sciences, Tehran, Iran

**Keywords:** natural killer cells, niche, adipose tissue, immune escape, natural cytotoxicity receptors

## Abstract

There are many types of leukocytes reside in subcutaneous adipose tissue (SAT), and among them, natural killer cells (NKs) comprise a major part. We show that the NKs that reside in the SAT (adipose tissue-derived NK cells; ADNKs) of the abdominal region found with phenotypic differences from the NKs circulating in the peripheral blood derived NK cells (PBNKs). In this survey, flow cytometry phenotyping was used to study the differences between the natural cytotoxicity receptor expression on ADNKs and PBNKs of both obese and lean persons. Also, their cytotoxicity and cytokine production patterns were evaluated. The activation experiments on isolated and expanded NKs with IL-2, IL-15, and IL-21 cytokines revealed the main population of the CD56^dim^ within the total ADNKs of obese persons has an under-expression of NKp30 and NKp44 despite the unchanged levels of NKG2D. The data suggest the suppressive condition of the adipose tissue niche on the NKs response against sensitive major histocompatibility complex class I non-expressing neoplastic cells. As the NKs are the first line of the body’s defense vs tumor formation, this change may lead to the development of transformed cells into the tumors.

## Introduction

According to the WHO, nowadays there are more than a 1.9 billion overweight people worldwide and of these, over 600 million are obese (1). This situation elevates the prevalence of certain cancers and immunological disorders. The obesity induces hypertrophy of adipocytes and limits the access to oxygen and nutrients (2). The resulting stress leads to the activation of pro-inflammatory cells in adipose tissue (3). Some various immune cell subsets, including macrophages, eosinophils, natural killer cells (NKs), NKT cells, and T cells, are important in maintaining adipose tissue homeostasis (4–7).

Natural killer cells are able to eliminate malignant cells with a strong regulatory role, by modulating both innate and adaptive immunity *via* crosstalk with cells of the innate and the adaptive immune system (8). Conventional NKs originates from committing progenitors of the bone marrow. They go through a maturation process and in this way NKs are influenced by the transcriptional network that guides them for phenotypic changes into two functionally distinct subpopulations, the CD56^dim^, and a minority subpopulation of CD56^bright^ cells (9). They are controlled by soluble factors such as chemokines, cytokines, and other secreted ligands of NKs receptors. Due to that, there is an extreme diversity of NKs repertoire over time (10). The receptors considered prevailing are those associated with receptor tyrosine-based activation motif (ITAM) bearing signaling molecules such as NKp_30_, NKp_44_, and NKp_46_ natural cytotoxicity receptors (NCRs) (11). A difficulty in understanding the basis of the NKs unresponsiveness in many cases, especially against tumor evasion related to the inexpression of activating receptors or existence of the inhibitory signals that have not been identified yet. So, the microenvironmental niches could dramatically affect the generation and function of distinct NKs subsets. Illuminating the platforms governing NKs function is a wonder, as they usually contravene the principle of other lymphocytes status.

Some of the NKs receptors protect normal cells by recognizing major histocompatibility complex class I (MHC-I) molecules. Cells expressing low levels of the MHC-I (usually cancer cells) are attacked by the NKs (12), but, other activating receptors on NKs such as lectin-like NKG2D, a member of the NKG2s family can activate the NKs without MHC-I ligands (13). While most of the activating receptors are specific for non-MHC-I ligands that are expressed on both normal and malignant cells, most of the inhibitory receptors are specific for the MHC-I ligand (14). Killer cell immunoglobulin-like receptors (KIRs) family includes the major inhibitory receptors (KIR3DL1-3, KIR2DL1-3, and KIR2DL5) which recognize the MHC-I molecules (HLA-A, B, and C), specifically (14, 15), while those of the NKG2s family recognize MHC-I signal sequences bound to the non-classical MHC molecule HLA-E (15). Both the KIRs and NKG2s families are composed of activating/inhibitory molecules that regulate the NKs function; however, they are originated from distinct gene families. Even the differences in a family are evident as the cytolytic activity of the NKs is inhibited by the NKG2A and activated by the NKG2C molecules (14).

Natural killer cells could enter to the peripheral tissues and recognize the stressed cells. They could react to the adipose stress and cause inflammation and even insulin resistance (16). In this way, several mechanisms such as insulin-like growth factor, adipocytes derived hormones (i.e., leptin), steroid hormones, chemokines, and adipokines may alter the function of NKs in this niche (17). Also, mesenchymal stem cells (MSCs) that comprise about 1% of the adipose niche have an immunomodulatory role and could suppress immune reactions *in vivo* in a non-HLA-restricted manner. The MSCs inhibit several functions of T lymphocytes (18, 19) and dendritic cells (DCs) (20). Their immunomodulatory role directed scientists for using MSCs to treat graft vs host disease after allogeneic hematopoietic transplantation (21). Some studies show the activated phenotype of NKs in visceral adipose tissue (22) suggesting their role in adipose tissue inflammation in metabolic disease. They produce pro-inflammatory cytokines, notably tumor necrosis factor alpha (TNFα) and interferon gamma (IFNγ), and regulate macrophages to promote insulin resistance in obesity (16, 23). Although there are some data on visceral adipose tissue role on NK cell phenotypic alteration, we did start first to know about the subcutaneous adipose tissue (SAT) niche for NKs behavior and molecular changes. Here, we have confirmed the alteration of the CD56^dim^ NKs NCRs especially NKp30 and NKp44, with respect to their cytotoxicity potential against malignant cells. This finding can open a new window to our knowledge of the increased rate of cancers in obese persons (11) such as esophageal adenocarcinoma, breast and colon cancers (24), and solving the problems of how these NKs will affect human diseases.

## Materials and Methods

### Sampling and NK Cell Isolation

This study was approved by the ethical committee of the Tehran University of Medical Sciences, Tehran, Iran. The adipose tissue was derived from normal obese volunteers aged between 18 and 70 years *via* abdominal lipoaspiration process. They were coming to the six of our collaborating clinics in Tehran for cosmetic reasons [52 persons with BMI ≥ 30 kg/m^2^ (94% female) and 18 persons with BMI ≤ 25 kg/m^2^ (44% female)]. All cases that entered into the study were negative for HIV, HBV, HCV, HTLV-1, and *Mycobacterium tuberculosis* infectious agents. Also, all of the volunteers provided the informed consent. The tumescent technique was used for lipoaspiration, and it was same for both groups of samples. Also, the peripheral blood (PB) was obtained from these volunteers for the comparison between peripheral blood-derived NK cells (PBNKs) and adipose tissue-derived NK cells (ADNKs). SAT (20 ml for obese persons and only 10 ml for non-obese persons) was subjected to collagenase digestion, as described previously (25) to obtain the stromal vascular fraction (SVF). Mononuclear cells were isolated from SVF and also PB by Ficoll Paque centrifugation. CD3^+^ cells were eliminated by negative selection (Miltenyi Biotec, Gladbach, Germany) based on the manufacturer’s instructions. CD56^+^ cells were isolated using anti-CD56 microbeads (Miltenyi Biotec). CD56^dim^ and CD56^bright^ subpopulations were separated with a BD Bioscience cell sorter (BD Bioscience, USA). The purity of CD56^dim^ NKs was 99%, and it was confirmed with the BD Bioscience cell sorter.

### Immunophenotyping Tests

For immunophenotyping assays, the panel of monoclonal antibodies (mAbs) against human CD3, CD4, CD16, CD56, Granzyme B, CD158b (KIR2DL2/3) (FITC conjugated; Biolegend, San Diego, CA, USA), NKp30 (CD337), NKp44 (CD336), NKp46 (CD335), NKG2D (CD314) and CD244 (2B4) (PEcy5 conjugated; Becton, Dickinson and Company; Mountain View, CA, USA), and CD107a (FITC conjugated; Pharmingen, San Diego, CA, USA) were prepared and all of them were used based on the manufacturer’s protocol. Surface marker staining was performed as follows: the cells were washed twice with FACS buffer [PBS with 0.1% sodium azide (NaN3; Sigma-Aldrich, USA) and 0.1% bovine serum albumin (Sigma-Aldrich, USA)] and then centrifuged for 10 min at 400 *g*. The mentioned mAbs was added in the dark room at 4°C using at 1:200 concentrations. Isotype-matched irrelevant mAbs was used to define non-specific staining. In the next step, the NKs were incubated at 4°C for 90 min and washed twice in FACS buffer. The samples were analyzed by the FACSCalibur and CellQuest analysis software (Becton, Dickinson and Company, USA). Data were gated on FITC-positive cells as described previously (26). After exclusion of non-viable cells using vital dye, a large forward and side-scatter gate was used to include viable cells, followed by gating on the CD45 marker, followed by gating on cell populations of interest.

### Expansion and Activation of Isolated ADNKs and PBNKs

After isolation and sorting out CD56^dim^ NKs, the process was followed by expansion of cells in XVIVO-20 medium (Lonza, Barcelona, Spain) supplemented with 10% fetal bovine serum (FBS, Gibco, UK), 1,000-IU/ml IL-2 (Promokine, Germany), 10 ng/ml IL-12 (Promokine, Germany), 50 ng/ml IL-15 (Promokine, Germany), and finally 10 ng/ml IL-21 (Promokine, Germany) for activation of cells. The process lasts for about 7 days.

### Tumor Cell Lines

The human neuroblastoma (NB) cell lines of SK-NSH (ATCC-HTB11TM) and CHLA255 (DSMZ-Germany), both were negative for MHC-I have been used in this study. They were cultivated in RPMI supplemented with 50 µg/ml penicillin–streptomycin antibiotics, 2 mM l-glutamine and 5% FBS at 37°C with 5% CO_2_. The cell viability was determined by trypan blue (Merck, Germany) staining.

### Cytokines Quantitation

For cytokine release measurement, the NKs from adipose tissue and PB were cultured in 96-well plates (10^5^ NKs per well) and then were subjected to IL-2 (10 ng/ml) and IL-15 (50 ng/ml). After 4 days, the viability of NKs was determined and the supernatant of all groups was collected by centrifugation (the samples were stored at −70°C until they were subjected to ELISA). The IFNγ and TNFα were quantified by an ELISA kit (Abcam, Cambridge, MA, USA) based on the manufacturer’s instruction. The amounts of these cytokines are expressed as ng/ml of NKs media.

### Real-time PCR Analysis

Total RNA from ADNKs and PBNKs of both sources was prepared by using Trizol and RNeasy Mini Kit (Qiagen, Germany) as described previously (27). The extracted RNA was amplified, after which cDNA was generated by using an Advantage RT-PCR kit (Clontech, USA). Gene expression levels were determined by real-time RT-PCR (ABI). The probes used in the experiments were for TNFα (Mm00443258_m1), IL-1b (Mm00434227_g1), IL-6 (Mm00446190_m1), IFNγ (Mm01168134_m1), IL-10 (Mm00439614_m1), IL-15 (Mm00434310_m1), IL-12a (Mm00434169_m1), and IL8 (Mm00434226_m1). GAPDH (Mm99999915_g1) served as a housekeeping control gene (ThermoFisher, USA).

### Cytotoxicity Assay

Natural killer cells (10^5^ cells per well) were incubated with NB target cells at ratios of 1:1, 3:1, and 5:1, respectively, for 4 h in 96-well plates with the 200 µl medium per well. NK-mediated cytotoxicity was analyzed by lactate dehydrogenase assay kits (Roche, Mannheim, Germany). The procedures of the assay were followed according to the manufacturer as previously described (28). Briefly, the culture medium was collected after treatment for the assay. The reaction underwent in the dark for 30 min before measurement. Changes in the absorbance were measured at 492 nm by a multiplate reader (Dynex, UK). Results were expressed as the percentage of control. Also, evaluation of the CD107a was performed to detect the degranulation capability of NKs as described in the Section “Immunophenotyping Tests.”

### Statistical Analysis

Student’s *t*-test was employed to determine the differences in NCR receptor surface expression on NK cells before and after IL-2 expansion in all groups. The results were analyzed using one-way ANOVA to determine the significance of observed differences. Data are expressed as means ± SEM, and the *p*-values of <0.05 were significant.

## Results

### ADNKs vs PBNKs

**Figure 1:**
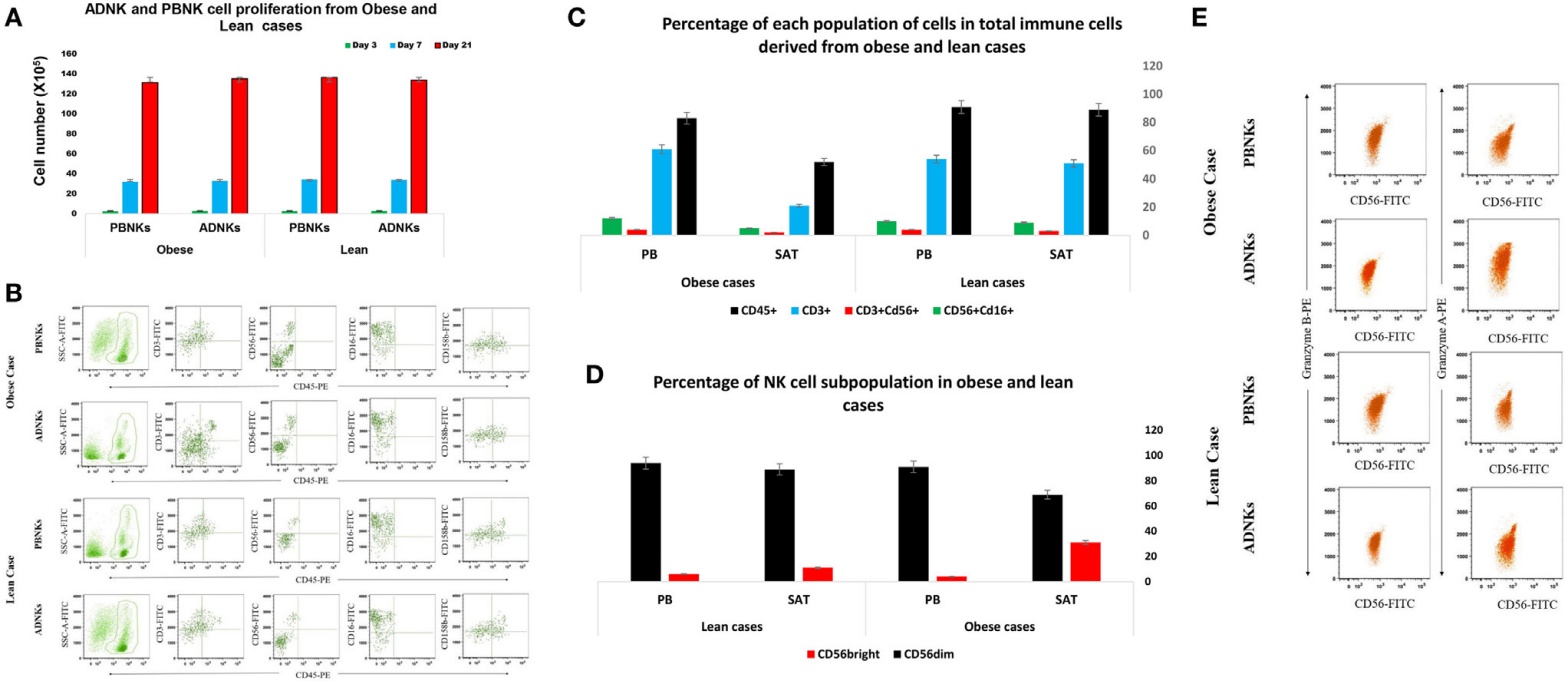
Circulating natural killer cells (NKs) in PB was similar in number both in obese (*N* = 52) and lean individuals (*N* = 18). The niche of NKs did not affect their proliferation rate after 3 (green bar), 7 (blue bar), and 21 (red bar) days of culture (*p = 0.008) **(A)**. Heterogeneous expression of the adhesion molecule CD56, Fc receptor CD16, and CD158b in ADNKs and PBNKs in a selected healthy obese person and non-obese persons. The samples were simultaneously stained with FITC-labeled anti-CD56 monoclonal antibody (detecting CD56^bright^ and CD56^dim^ NK subsets). Data were gated on FITC-positive cells and the cells were analyzed on the basis of forward- and side-scatter parameters and CD56 fluorescence **(B)**. The percentage of CD45^+^, CD3^+^, CD56^+^, and CD16^+^ cells in obese and lean cases **(C)**. The pattern of CD56 phenotypes in NKs from both sources that shows the difference between these two subpopulations of NKs including ADNKS and PBNKs (*p* = 0.005) **(D)**. Represented flow cytometry dot plots of one subject about the granzyme A and granzyme B content of NKs in all groups **(E)**. (All the data obtained from 70 samples, and the data were statistically significant.) PB, peripheral blood; PBNKs, peripheral blood-derived NK cells; SAT, subcutaneous adipose tissue; ADNKs, adipose tissue-derived NK cells.

**Figure 2:**
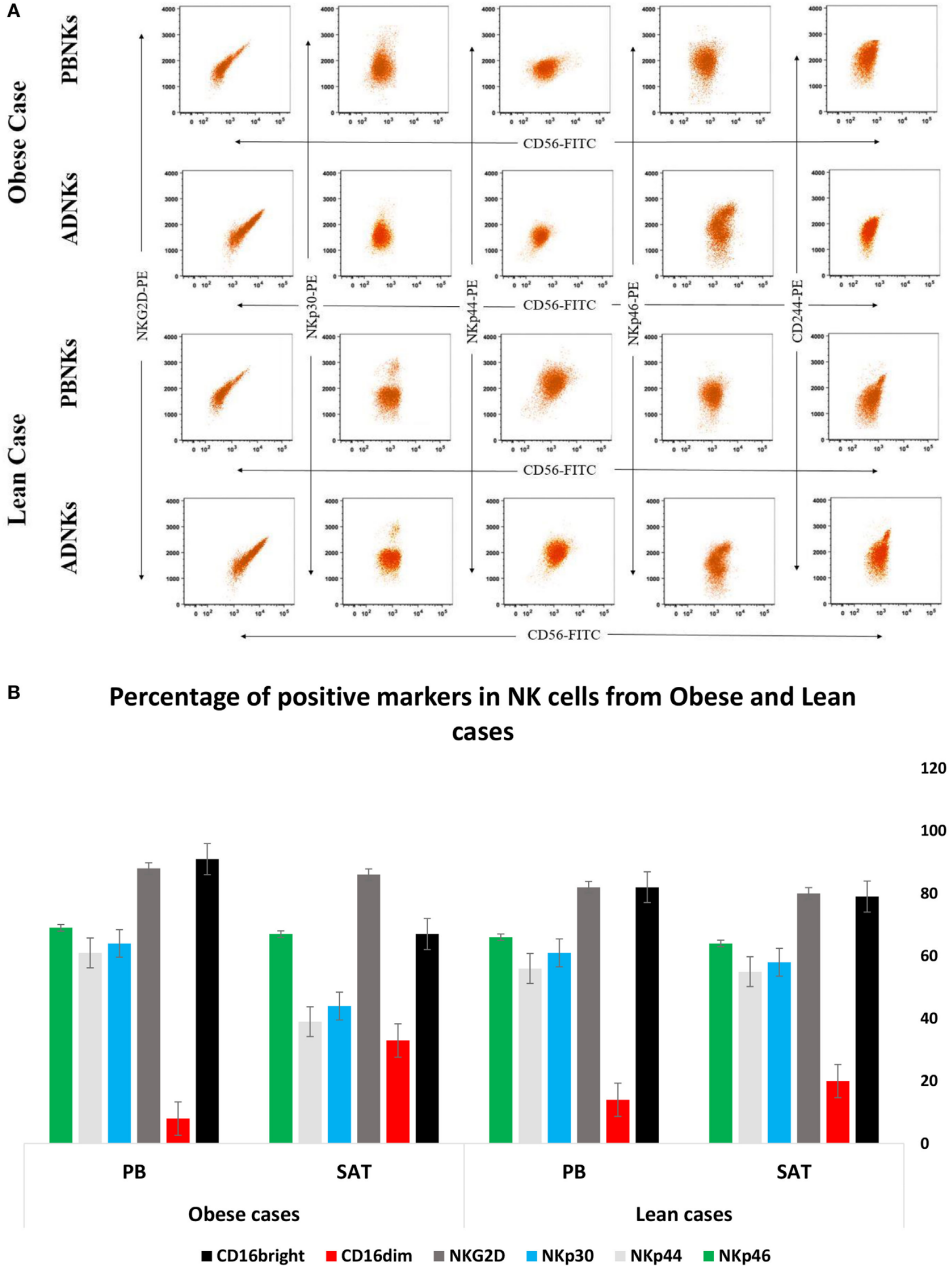
The enriched population of CD56^+^ was stained with PE-labeled natural cytotoxicity receptors (NCRs), including NKp30, NKp44, NKp46, and NKG2D and analyzed by flow cytometry **(A)**. The graph represents the quantified number of NCRs from all 70 samples in percentage of each positive receptor/marker on the natural killer cells (NKs). The data were statistically significant (*p = 0.005) **(B)**. PB, peripheral blood; PBNKs, peripheral blood derived NK cells; SAT, subcutaneous adipose tissue; ADNKs, adipose tissue-derived NK cells.

No significant differences were detected between numbers of proliferating NKs from adipose tissue in comparison with the NKs from PB (Figure 1A). ADNKs have been distinguished by the differentially expressed surface markers. Flow cytometry was used to characterize the expression of the adhesion molecule CD56, Fc receptor CD16, granzyme B, and the CD158b in both ADNKs and PBNKs in healthy, lean (BMI ≤ 25) and obese persons (BMI ≥ 30) (Figure 1B). In obese and lean persons, CD45^+^ cells and CD3^+^ cells comprised a greater percentage of total PB mononuclear cells than the SVF of adipose tissue, while frequencies of CD3^+^CD56^+^ cells and CD3^−^CD56^+^ (NKs) were same between them, but the NKs that reside in the SAT of the abdominal region found with different phenotype from NKs circulating in the PB (Figure 1C). There is a slight difference between CD56^bright^ and CD56^dim^ NKs belongs to the ADNKS and PBNKs between obese and normal weight individuals (*p* < 0.05; Figure 1D). The analysis of all 52 ADNK samples from obese persons confirmed a slight shift to CD56^bright^ phenotype (*p* < 0.05). In lean persons, the frequencies of CD56^dim^ populations within the total NKs population were decreased in ADNKs and increased in PBNKs, respectively. Granzyme B and perforin were expressed at similar levels in CD56^dim^ ADNKs and PBNKs, whereas granzyme A was twofolds overexpressed in ADNKs vs the CD56^dim^ PBNK cells (Figure 1E). Expression levels of the NKs activation marker NKG2D were similar in all groups, but the levels of NKp30 and NKp44 decreased within the ADNKs population of obese compared to lean persons (Figure 2A). We did not face any defect in NKp46 expression between all groups. After expansion and activation of NKs, we could see the alteration in important NCRs expression of ADNKs especially for NKp30 and NKp44 (Figure 2A) at the same treatment conditions by cytokines. The quantified diagram of NCRs expression could be seen in Figure 2B (*p* < 0.05).

### Transcriptome Shift in ADNKs vs PBNKs

**Figure 3:**
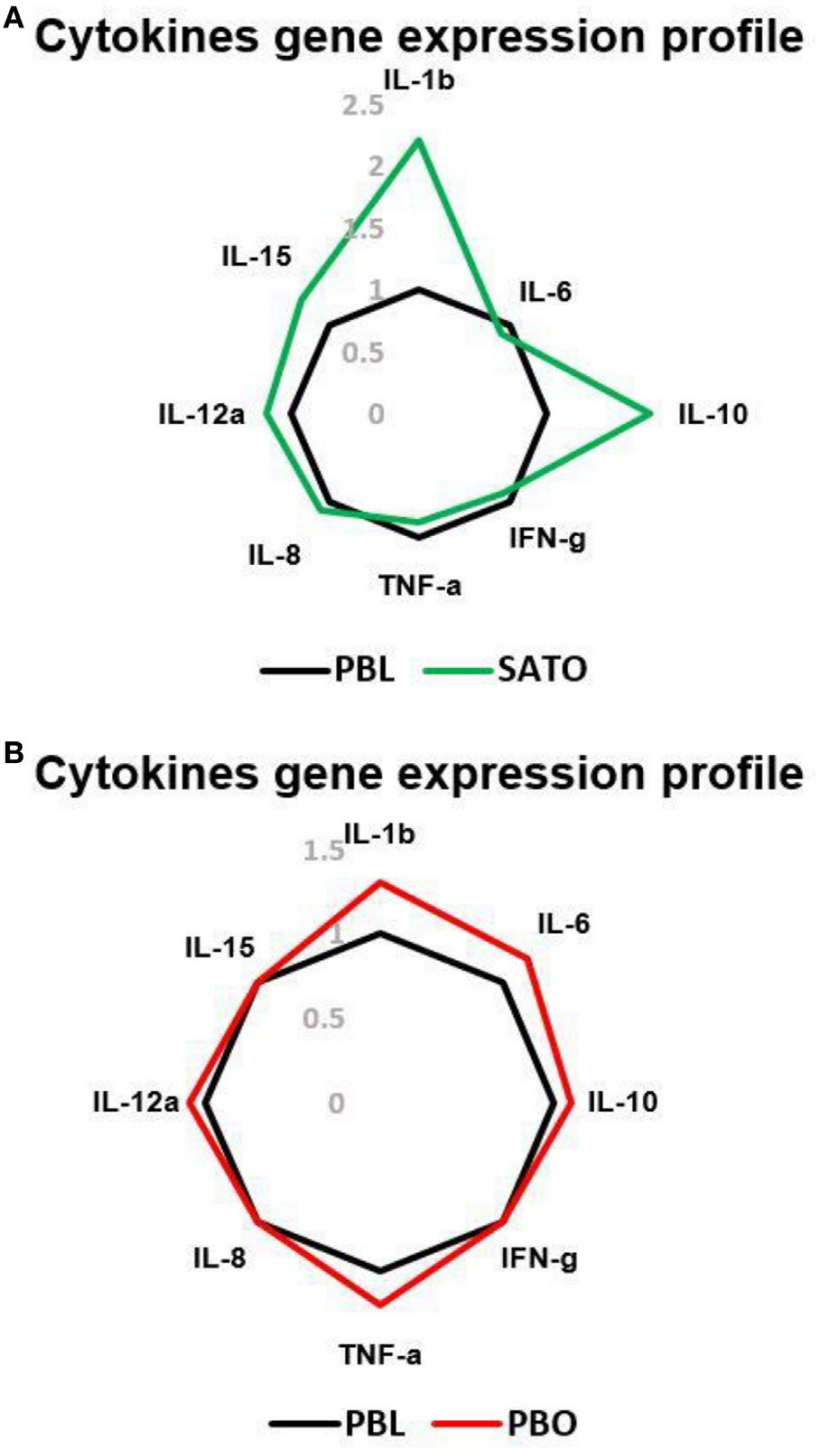
The gene expression profile of IL-1b, IL-6, IL-8, IL-10, IL-12a, IL-15, interferon gamma (IFNγ), and tumor necrosis factor alpha (TNFα) in subcutaneous adipose tissue-derived **(A)** and peripheral blood (PB) **(B)** of total natural killer cells (NKs) in obese and lean cases *via* qRT-PCR. The levels of these cytokines in obese persons are compared to the PB total NK cells of lean cases as the control. PBL, peripheral blood of lean cases; PBO, peripheral blood of obese cases; SATO, subcutaneous adipose tissue derived of obese cases.

RNA from SVFs isolated from SAT in the abdominal region of obese persons was studied with qRT-PCR. SVF was a source of IFNγ, TNFα, IL-10, IL-8, and cytokines. So, SVF is a source of inflammatory cytokines (Figure 3A). It has been known that PB CD56^bright^CD16^−^ NK cells produce larger amounts of cytokines compared to CD56^low^CD16^+^ NKs (29). Thus, we investigated whether subcutaneous ADNK cells could produce cytokines such as IFNγ, early upon activation. As shown in Figure 3A, IL-2 could not promptly induce IFNγ production by ADNKs from obese cases. As previously described for CD56^high^CD16^−^ NK cells, isolated from PB (29), more NK cells produced IFNγ upon exposure to IL-2 and IL-12 cytokines compared with stimulations with the individual cytokines. There was a decreased level of IL-6, IFNγ, and TNFα from ADNKs (Figures 3A,B). The levels of these cytokines could be a parallel index to the NCRs expression for NKs unresponsiveness to neoplastic cell cultures.

### Cytotoxicity Assay and Cytokine Secretion

**Figure 4:**
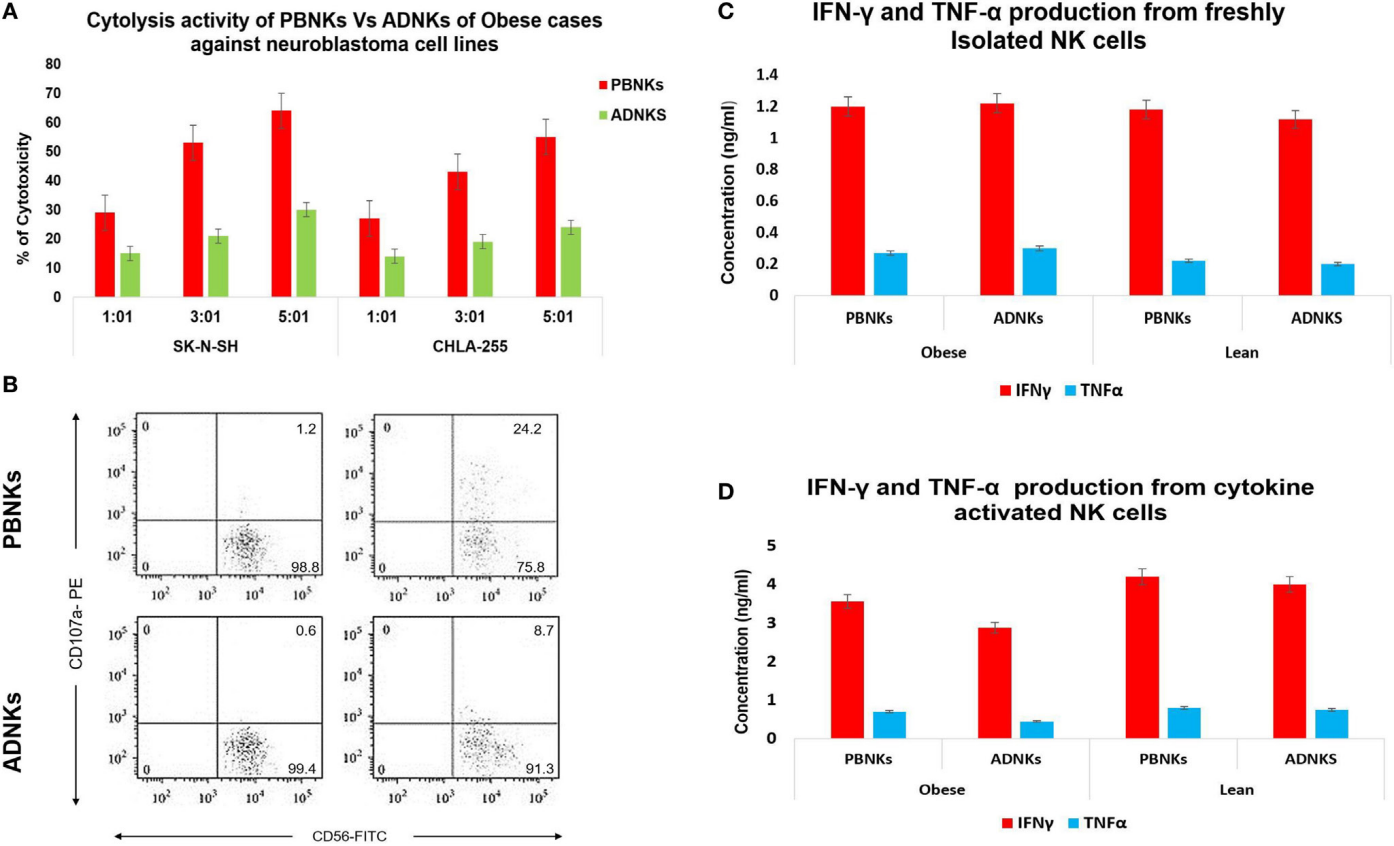
The cytotoxicity assay of ADNKs vs PBNKs on the SK-N-SH and CHLA-255 neuroblastoma cell lines in the 1:1, 3:1, and 5:1 ratios **(A)**. Granzyme degranulation marker CD107a expression of natural killer cells (NKs) before (left side) and after (right side) NB cell stimulation was measured. It shows a significant correlation between degranulation amounts of ADNKs of the study groups (*p* = 0.005). The profiles show CD107a surface staining of lysosomal proteins (sideward scatter), as indicated on CD3^−^CD56^+^ (forward scatter) NK cells. The percentage of CD107a^+^ cells (up right), CD56^+^ cells (down right), and CD3^+^, CD4^+^ cells (left side) is indicated on each plot **(B)**. The data show the expanded NKs after stimulation with IL-21 can induce activation signaling to PBNKs and ADNKs. ELISA data have shown no changes in cytokine production in the absence of any stimulation on NK cells **(C)** but there is a decreased level of both interferon gamma (IFNγ) and tumor necrosis factor alpha (TNFα) in ADNKs when compared to the normal PBNKs **(D)**. All data were statistically significant (*p* < 0.05, each experiment was performed in triplicate). PBNKs, peripheral blood-derived NK cells; ADNKs, adipose tissue-derived NK cells.

Previous studies demonstrated that the ability of human NK cells to kill MHC-I non-expressing neoplastic targets correlates with the surface density of the NCRs (13, 30). In line with the results obtained in ADNKs from obese cases, they displayed poor cytolytic activity against the target cells. On the contrary, ADNKs from lean cases displayed a cytolytic activity comparable in magnitude to their PBNKs (Figure 4A). The neoplastic cells induce regular CD56^dim^ NKs to express high levels of the NKp44 receptor, but in the ADNKs there was no significant change of this important NCR (*p* < 0.05; Figure 2B). Also, the flow cytometry analysis demonstrated decreased levels of CD107a after NKs coculture with neoplastic cell line (Figure 4B). When the degranulation of NKs was investigated by the expression pattern of CD107a, the basal expression level of CD107a on ADNKs from lean persons was about 1.2% and this amount for obese donors was less than 0.6% that shows 50% decrease. The differences between two populations arise when the NKs are subjected to target NB cells for cytotoxicity assay (*p* < 0.05; Figure 4B). Unlike perforin, granzyme B was expressed at similar levels in ADNKs and PBNKs, whereas granzyme A was twofold overexpressed in ADNKs vs the PBNK (Figures 1E and 4B). To investigate potential mechanisms underlying the cytotoxic effects of NK cells, we measured production of intracellular cytokines by NK cells in all groups. When NK cells were not challenged with tumor cells, their source in our groups had no effect on cytokine production by NK cells (Figure 4C). When NK cells were stimulated with IL-21 before their adjoining to target cells, an adipose niche in obese persons significantly inhibited NK cell’s ability to lyse neoplastic cells (Figure 4A). The ADNKs from obese cases have a decreased production of intracellular IFNγ (Figure 4D) upon tumor challenge. In line with transcription level data, the similar situation happened for TNFα production (Figure 4D) and we demonstrated the decreased level of its production in NK cells derived from adipose tissue niche in obese cases.

## Discussion

In this study, we provide the phenotypic characteristics of NK cells reside in subcutaneous abdominal fat in obese persons. Also, it is important to mention that the visceral adipose tissue completely differs from the subcutaneous fat counterpart in behavior and gene expression profile (31). Epidemiological evidence indicate that obesity is responsible for more than 15% of all deaths for persons beyond 40 years of age in the United States (32). NK cells are a population of lymphocyte that enters in adipose tissue and reside there for their whole life. As NKs are the first line of innate defense vs tumor formation, the phenotypic changes in the surface markers, KIRs, and NCR expression may lead to the progress of transformed cells into a tumor. On the other hand, the impaired NKs function might cause autoimmune disease and persistence of autoimmunity. NK cell localization in the other tissues might be a prerequisite for maturation into the classical cytolytic phenotype, found predominantly in PB.

There are some reports that show the dysfunction of NKs caused by hormones and cytokines. In 2006, Kim et al. found that adiponectin is a negative regulator of NKs cytotoxicity (33), but there are no reports on the mechanism underlying this dysfunction. Our experiment was unable to detect the significant difference in the number of NKs between obese and lean cases (Figure 1A), but the shift of ADNKs to CD56^bright^ phenotype was obvious and our findings show a marked reduction in the function of NKs in obese cases. The expression of CD16 was reduced slightly in ADNKs of obese cases, and there was a significant difference between ADNKs and PBNKs groups (Figure 1B). In 2004, Dovio et al. investigated NKs activity in patients with uncomplicated obesity with an average BMI of 36. Our study confirms their findings as we found that obese subjects with no metabolic complications had similar NK cell levels to lean controls. They believe it was only metabolically unhealthy obese subjects which make up 80% of the obese population, have reduced NK cell levels, and altered phenotype (34). In our experiment that conducted on the healthy individuals, the cytotoxicity tests on the NB cell lines resulted in the poor cytolysis activity of ADNKs in comparison with PBNKs (Figure 4A). By increasing the body mass index, more and more NKs will reside in this niche and they would be non-functional for their entire life.

The responses to the question that why entrapped NKs in the adipose tissue lose their cytolytic effect on neoplastic cells may be found in the MSCs interaction with the NKs. Yet, so far 15 years that researchers found a large population of MSCs in the adipose tissue (ADSCs) (35). Probably these stromal cells could influence the niche of abdominal adipose tissue too, especially by exerting their immunomodulatory function (36) directly by cell–cell contact or *via* production of cytokines such as IL-10, IL-6, and TGF-β (37). Many piece of evidence are emphasizing on the stronger immunomodulation activity of ADSCs compared to bone marrow-MSCs (BMSCs). In 2009, Ivanova-Todorova et al. showed that ADSCs are more potent suppressors of DCs compared to BMSCs (38). Spaggiari and colleagues focused on the ADSCs secreted indoleamine 2,3-dioxygenase and prostaglandin E2 role in the suppression of proliferation, cytolysis, and cytokine production in the NKs (39). In this way, Selmani et al. (40) confirmed that human leukocyte antigen-G5 secretion by MSCs is required to suppress T and NK cell function and to induce CD25^high^FOXP3^+^ regulatory T cells (40). An important change that detected in our flow cytometry analysis was related to the downregulation of Nkp30 and Nkp44 (Figure 2A). We know that NKp30 and NKp46 are constitutively expressed by all NK cells, while NKp44 is expressed only after activation. So, we detected the levels of Nkp44 in freshly isolated NKs and also after cytokine activation. In addition to the levels of NKp44, our results have been demonstrated the reduced levels of NKp30 receptors in ADNKs when compared with PB CD56^dim^ NKs (Figure 2B). The NKp44 suppression strongly contributes to the unresponsiveness of NKs against sensitive malignant cells that did not express MHC class I molecule (Figure 4A). The amount of Nkp46 expression was similar in ADNKs and PBNKs in both obese and lean persons (Figure 2B). As the gene coding for NKp46 is located in the leukocyte-receptor complex on human chromosome 19, while the genes for NKp44 and NKp30 are located on chromosome 6 (41) maybe this alteration happened only in one chromosome that belongs to Nkp30 and NKp44 NCRs. The NKp44 has unique features of several occasions including (1) its expression is closed to active NKs authoritative to initiate an immediate cytotoxic response (42), (2) its expression is responsible for killing of many tumor cells and also results in the release of cytotoxic granules, IFNγ, and TNFα (43), (3) it is also expressed on a subset of IFN-producing cells located in mucosal-associated lymphoid tissues (44). By downregulation of NKp44, tumor cells could induce NKs apoptosis *via* overexpression of Fas Ligands (45). NKp44 could be exploited and the tumor cells escape from the NKs recognition due to the changes in their niche. Our data show that the SAT in the abdominal region of obese persons provides this kind of microenvironment that induce these phenotypic changes in NKs (Figures 1B and 2A). However, we can see the similar expression of NKG2D levels in obese persons when compared with non-obese cases (Figure 2B). But, it has been reported by O’Rourke et al. that the adipose tissue NKs had an activated phenotype with increased levels of NKG2D (22). This increased expression of NKG2D may cause inflammation in obesity. Also, we have demonstrated the altered expression of granzyme B (Figure 1E) in stimulated NKs that might lead to NK-mediated death.

The cytokine secretion experiments showed decreased secretion levels of IFNγ and TNFα cytokines (Figure 4D). Recent studies show that MSCs cause a decrease in IFNγ secretion by NKs in coculture systems (46). We confirmed this situation for some HLA type I non-expressing neoplastic cells after cell to cell contact and/or coculture of PBNKs with ADMSCs (Data not shown). Some other studies have discussed the role of leptin that produced by adipocyte on the cytokine secretion by NKs.

Our findings provide preliminary data onto the relationship between abdominal adipose tissue and the NKs biology that undoubtedly influences the rate of malignant diseases or even metabolic disorders, especially in obese persons. The role of other cells and/or factors that affect NCRs under-expression is under investigation by our team and needs more evidence to be fully elucidated.

## Ethics Statement

All methods were carried out in the experiments with human cells in accordance with the ethical committee of the Tehran University of Medical School. All the protocols were approved by the ethics committee of Tehran University of Medical Sciences and Ministry of Health and Medical education. Also, all of the volunteers provided the informed consent.

## Author Contributions

AS-H, MB, RM, and JA performed the experiments; SM-T performed statistical analysis; AH designed the study; SM-T and AH contributed in writing the manuscript.

## Conflict of Interest Statement

The authors declare that the research was conducted in the absence of any commercial or financial relationships that could be construed as a potential conflict of interest.

## References

[1] Obesity and Overweight. (2017). Available from: http://www.who.int/mediacentre/factsheets/fs311/en/

[2] Khan T Muise ES Iyengar P Wang ZV Chandalia M Abate N Metabolic dysregulation and adipose tissue fibrosis: role of collagen VI. Mol Cell Biol (2009) 29:1575–91. 10.1128/MCB.01300-08 19114551 PMC2648231

[3] Hotamisligil GS Inflammation and metabolic disorders. Nature (2006) 444:860–7. 10.1038/nature05485 17167474

[4] Huh JY Park YJ Ham M Kim JB Crosstalk between adipocytes and immune cells in adipose tissue inflammation and metabolic dysregulation in obesity. Mol Cells (2014) 37(5):365–71. 10.14348/molcells.2014.0074 24781408 PMC4044307

[5] Lynch L Nowak M Varghese B Clark J Hogan AE Toxavidis V Adipose tissue invariant NKT cells protect against diet-induced obesity and metabolic disorder through regulatory cytokine production. Immunity (2012) 37:574–87. 10.1016/j.immuni.2012.06.016 22981538 PMC4991771

[6] Wu D Molofsky AB Liang HE Ricardo-Gonzalez RR Jouihan HA Bando JK Eosinophils sustain adipose alternatively activated macrophages associated with glucose homeostasis. Science (2011) 332:243–7. 10.1126/science.1201475 21436399 PMC3144160

[7] Ferrante AW The immune cells in adipose tissue. Diabetes Obes Metab (2013) 3:34–8. 10.1111/dom.12154 PMC3777665 24003919

[8] Shoae-Hassani A Hamidieh AA Behfar M Mohseni R Mortazavi-Tabatabaei SA Asgharzadeh S NK cell derived exosomes from NK cells previously exposed to neuroblastoma cells augment the antitumor activity of cytokine activated NK cells. J Immunother (2017) 40(7):265–76. 10.1097/CJI.0000000000000179 28622272 PMC7543683

[9] Robertson MJ Ritz J Biology and clinical relevance of human natural killer cells. Blood (1990) 76(12):2421–38. 2265240

[10] Bezman NA Kim CC Sun JC Min-Oo G Hendricks DW Kamimura Y The immunological genome project consortium molecular definition of the identity and activation of natural killer cells. Nat Immunol (2012) 13:1000–9. 10.1038/ni.2395 22902830 PMC3572860

[11] Renehan AG Tyson M Egger M Heller RF Zwahlen M. Body-mass index and incidence of cancer: a systematic review and meta-analysis of prospective observational studies. Lancet (2008) 371(9612):569–78. 10.1016/S0140-6736(08)60269-X 18280327

[12] Ljunggren HG Kärre K. In search of the ‘missing self’: MHC molecules and NK cell recognition. Immunol Today (1990) 11:237–44. 10.1016/0167-5699(90)90097-S 2201309

[13] Moretta A Bottino C Vitale M Pende D Cantoni C Mingari MC Activating receptors and coreceptors involved in human natural killer cell-mediated cytolysis. Annu Rev Immunol (2001) 19:197–223. 10.1146/annurev.immunol.19.1.197 11244035

[14] Fernández-Messina L Reyburn H Vales-Gomez M. Human NKG2D-ligands: cell biology strategies to ensure immune recognition. Front Immunol (2012) 3:299. 10.3389/fimmu.2012.00299 23056001 PMC3457034

[15] Dahlberg CIM Sarhan D Chrobok M Duru AD Alici E. Natural killer cell-based therapies targeting cancer: possible strategies to gain and sustain anti-tumor activity. Front Immunol (2015) 6:605. 10.3389/fimmu.2015.00605 26648934 PMC4663254

[16] Wensveen FM Jelencic V Valentic S Sestan M Wensveen TT Theurich S NK cells link obesity-induced adipose stress to inflammation and insulin resistance. Nat Immunol (2015) 16(4):376–85. 10.1038/ni.3120 25729921

[17] Tilg H Moschen AR. Adipocytokines: mediators linking adipose tissue, inflammation and immunity. Nat Rev Immunol (2006) 6(10):772–83. 10.1038/nri1937 16998510

[18] Meisel R Zibert A Laryea M Human bone marrow stromal cells inhibit allogeneic T-cell responses by indoleamine 2,3-dioxygenase mediated tryptophan degradation. Blood (2004) 103:4619–21. 10.1182/blood-2003-11-3909 15001472

[19] Ghannam S Pene J Moquet-Torcy G Jorgensen C Yssel H. Mesenchymal stem cells inhibit human Th17 cell differentiation and function and induce a T regulatory cell phenotype. J Immunol (2010) 185(1):302–12. 10.4049/jimmunol.0902007 20511548

[20] Spaggiari GM Abdelrazik H Becchetti F Moretta L. MSCs inhibit monocyte-derived DC maturation and function by selectively interfering with the generation of immature DCs: central role of MSC-derived prostaglandin E2. Blood (2009) 113(26):6576–83. 10.1182/blood-2009-02-203943 19398717

[21] Chen X Wang C Yin J Xu J Wei J Zhang Y. Efficacy of mesenchymal stem cell therapy for steroid-refractory acute graft-versus-host disease following allogeneic hematopoietic stem cell transplantation: a systematic review and meta-analysis. PLoS One (2015) 10(8):e0136991. 10.1371/journal.pone.0136991 26323092 PMC4554731

[22] O’Rourke RW Gaston GD Meyer KA White AE Marks DL. Adipose tissue NK cells manifest an activated phenotype in human obesity. Metabolism (2013) 62(11):1557–61. 10.1016/j.metabol.2013.07.011 24012153 PMC3809342

[23] Lee BC Kim MS Pae M Yamamoto Y Eberle D Shimada T Adipose natural killer cells regulate adipose tissue macrophages to promote insulin resistance in obesity. Cell Metab (2016) 23:685–98. 10.1016/j.cmet.2016.03.002 27050305 PMC4833527

[24] Calle EE Rodriguez C Walker-Thurmond K Thun MJ. Overweight, obesity, and mortality from cancer in a prospectively studied cohort of U.S. adults. N Engl J Med (2003) 348(17):1625–38. 10.1056/NEJMoa021423 12711737

[25] Amirkhani MA Mohseni R Soleimani M Shoae-Hassani A Nilforoushzadeh MA. A rapid sonication based method for preparation of stromal vascular fraction and mesenchymal stem cells from fat tissue. Bioimpacts (2016) 6(2):99–104. 10.15171/bi.2016.14 27525227 PMC4981255

[26] Abeles D McPhail MJ Sowter D Antoniades CG Vergis N Manakkat Vijay GK CD14, CD16 and HLA-DR reliably identifies human monocytes and their subsets in the context of pathologically reduced HLA-DR expression by CD14hi/D16neg monocytes: expansion of CD14hi/CD16pos and contraction of CD14lo/CD16pos monocytes in acute liver failure. Cytometry (2012) 81:823–34. 10.1002/cyto.a.22104 22837127

[27] Shoae-Hassani A Keyhanvar P Seifalian AM Mortazavi-Tabatabaei SA Ghaderi N Issazadeh K lambda Phage nanobioparticle expressing apoptin efficiently suppress human breast carcinoma tumor growth in vivo. PLoS One (2013) 8(11):e79907 10.1371/journal.pone.0079907 24278212 PMC3838365

[28] Smith SM Wunder MB Norris DA Shellman YG. A simple protocol for using a LDH-based cytotoxicity assay to assess the effects of death and growth inhibition at the same time. PLoS One (2011) 6(11):e26908. 10.1371/journal.pone.0026908 22125603 PMC3219643

[29] Fehniger TA Cooper MA Nuovo GJ Cella M Facchetti F Colonna M CD56^bright^ natural killer cells are present in human lymph nodes and are activated by T cell derived IL-2: a potential new link between adaptive and innate immunity. Blood (2003) 101:3052 10.1182/blood-2002-09-2876 12480696

[30] Sivori S Pende D Bottino C Marcenaro E Pessino A Biassoni R NKp46 is the major triggering receptor involved in the natural cytotoxicity of fresh or cultured human NK cells. Correlation between surface density of NKp46 and natural cytotoxicity against autologous, allogeneic or xenogeneic target cells. Eur J Immunol (1999) 29:1656–66. 10.1002/(SICI)1521-4141(199905)29:05<1656::AID-IMMU1656>3.0.CO;2-1 10359120

[31] Vohl MC Sladek R Robitaille J Gurd S Marceau P Richard D A survey of genes differentially expressed in subcutaneous and visceral adipose tissue in men. Obes Res (2004) 12:1217–22. 10.1038/oby.2004.153 15340102

[32] Masters RK Reither EN Powers DA Yang YC Burger AE Link BG. The impact of obesity on US mortality levels: the importance of age and cohort factors in population estimates. Am J Public Health (2013) 103:1895–901. 10.2105/AJPH.2013.301379 23948004 PMC3780738

[33] Kim KY Kim JK Han SH Lim JS Kim KI Cho DH Adiponectin is a negative regulator of NK cell cytotoxicity. J Immunol (2006) 176(10):5958–64. 10.4049/jimmunol.176.10.5958 16670304

[34] Dovio A Caramello V Masera RG Sartori ML Saba L Tinivella M Natural killer cell activity and sensitivity to positive and negative modulation in uncomplicated obese subjects: relationship to leptin and diet composition. Int J Obes Relat Metab Disord (2004) 28:894–901. 10.1038/sj.ijo.0802639 15208649

[35] Zuk PA Zhu M Mizuno H Huang J Futrell JW Katz AJ Multilineage cells from human adipose tissue: implications for cell-based therapies. Tissue Eng (2001) 7(2):211–28. 10.1089/107632701300062859 11304456

[36] Meisel R Zibert A Laryea M Gobel U Daubener W Dilloo D Human bone marrow stromal cells inhibit allogeneic T-cell responses by indoleamine 2,3-dioxygenase-mediated tryptophan degradation. Blood (2004) 103(12):4619–21. 10.1182/blood-2003-11-3909 15001472

[37] Elman JS Li M Wang F Gimble JM Parekkadan B A comparison of adipose and bone marrow-derived mesenchymal stromal cell secreted factors in the treatment of systemic inflammation. J Inflamm (Lond) (2014) 11:1 10.1186/1476-9255-11-1 24397734 PMC3895743

[38] Ivanova-Todorova E Bochev I Mourdjeva M Dimitrov R Bukarev D Kyurkchiev S Adipose tissue-derived mesenchymal stem cells are more potent suppressors of dendritic cells differentiation compared to bone marrow-derived mesenchymal stem cells. Immunol Lett (2009) 126:37–42. 10.1016/j.imlet.2009.07.010 19647021

[39] Spaggiari GM Capobianco A Abdelrazik H Becchetti F Mingari MC Moretta L. Mesenchymal stem cells inhibit natural killer-cell proliferation, cytotoxicity, and cytokine production: role of indoleamine 2,3-dioxygenase and prostaglandin E2. Blood (2008) 111:1327–33. 10.1182/blood-2007-02-074997 17951526

[40] Selmani Z Naji A Zidi I Favier B Gaiffe E Obert L Human leukocyte antigen-G5 secretion by human mesenchymal stem cells is required to suppress T lymphocyte and natural killer function and to induce CD4+CD25highFOXP3+ regulatory T cells. Stem Cells (2008) 26:212–22. 10.1634/stemcells.2007-0554 17932417

[41] Seidel E Glasner A Mandelboim O. Virus-mediated inhibition of natural cytotoxicity receptor recognition. Cell Mol Life Sci (2012) 69:3911–20. 10.1007/s00018-012-1001-x 22547090 PMC11115132

[42] Cantoni C Bottino C Vitale M Pessino A Augugliaro R Malaspina A NKp44, a triggering receptor involved in tumor cell lysis by activated human natural killer cells, is a novel member of the immunoglobulin superfamily. J Exp Med (1999) 189(5):787–96. 10.1084/jem.189.5.787 10049942 PMC2192947

[43] Vitale M Bottino C Sivori S Sanseverino L Castriconi R Marcenaro E NKp44, a novel triggering surface molecule specifically expressed by activated natural killer cells, is involved in non-major histocompatibility complex-restricted tumor cell lysis. J Exp Med (1998) 187:2065–72. 10.1084/jem.187.12.2065 9625766 PMC2212362

[44] Spits H Artis D Colonna M Diefenbach A Di Santo JP Eberl G Innate lymphoid cells – a proposal for uniform nomenclature. Nat Rev Immunol (2013) 13(2):145–9. 10.1038/nri3365 23348417

[45] Poggi A Massaro AM Negrini S Contini P Zocchi MR. Tumor-induced apoptosis of human IL-2-activated NK cells: role of natural cytotoxicity receptors. J Immunol (2005) 174(5):2653–60. 10.4049/jimmunol.174.5.2653 15728472

[46] Shi Y Su J Roberts AI Shou P Rabson AB Ren G. How mesenchymal stem cells interact with tissue immune responses. Trends Immunol (2012) 33(3):136–43. 10.1016/j.it.2011.11.004 22227317 PMC3412175

